# The Use of Tissue Concentrations of Biological and Small-Molecule Therapies in Clinical Studies of Inflammatory Bowel Diseases

**DOI:** 10.3390/pharmaceutics16121497

**Published:** 2024-11-22

**Authors:** Ahmed B. Bayoumy, Luc J. J. Derijks, Bas Oldenburg, Nanne K. H. de Boer

**Affiliations:** 1 Department of Gastroenterology and Hepatology, Amsterdam University Medical Center, 1105 AZ Amsterdam, The Netherlands; khn.deboer@amsterdamumc.nl; 2 Amsterdam Gastroenterology Endocrinology Metabolism Research Institute, 1105 AZ Amsterdam, The Netherlands; 3 Department of Clinical Pharmacy & Pharmacology, Máxima Medical Centre, 5631 BM Eindhoven, The Netherlands; 4 Department of Clinical Pharmacy & Toxicology and NUTRIM, School of Nutrition and Translational Research in Metabolism, Maastricht University Medical Centre, 6229 HX Maastricht, The Netherlands; 5 Department of Gastroenterology and Hepatology, University Medical Center Utrecht, 3584 CX Utrecht, The Netherlands

**Keywords:** inflammatory bowel disease, tissue concentrations, infliximab, ustekinumab, vedolizumab, methotrexate, tofacitinib

## Abstract

**Abstract:** The introduction of biological therapies has revolutionized inflammatory bowel disease (IBD) management. A critical consideration in developing these therapies is ensuring adequate drug concentrations at the site of action. While blood-based biomarkers have shown limited utility in optimizing treatment (except for TNF-alpha inhibitors and thiopurines), tissue drug concentrations may offer valuable insights. In antimicrobial therapies, tissue concentration monitoring is standard practice and could provide a new avenue for understanding the pharmacokinetics of biological and small-molecule therapies in IBD. Various methods exist for measuring tissue concentrations, including whole tissue sampling, MALDI-MSI, microdialysis, and fluorescent labeling. These techniques offer unique advantages, such as spatial drug-distribution mapping, continuous sampling, or cellular-level analysis. However, challenges remain, including sampling invasiveness, heterogeneity in tissue compartments, and a lack of standardized bioanalytical guidelines. Drug pharmacokinetics are influenced by multiple factors, including molecular properties, disease-induced changes in the gastrointestinal tract, and the timing of sample collection. For example, drug permeability, solubility, and interaction with transporters may vary between Crohn’s disease and ulcerative colitis. Research into the tissue concentrations of drugs like anti-TNF agents, ustekinumab, vedolizumab, and tofacitinib has shown variable correlations with clinical outcomes, suggesting potential roles for tissue concentration monitoring in therapeutic drug management. Although routine clinical application is not yet established, exploring tissue drug concentrations may enhance understanding of IBD pharmacotherapy.

## 1. Introduction

The introduction of biological therapies in the 1990s has significantly changed the management of inflammatory bowel disease (IBD) [1]. A key question for assessing the efficacy of new biological and small-molecule therapies during drug development is whether drug concentrations at the site of action are adequate to elicit a pharmacological effect. Given the disappointing performance of most blood-based biomarkers (except for TNF-alpha inhibitors and thiopurines) in optimizing treatment strategies, it might be worthwhile to explore the potential role of tissue concentrations of biological and small-molecule therapies in this setting. Tissue concentrations are widely used in antimicrobial therapies [2,3]. In IBD, acquiring knowledge of tissue concentrations might open new avenues to the understanding of drug pharmacokinetics, and subsequently enable therapeutic drug monitoring of biological and small-molecule therapies. In this commentary, we would like to provide the pearls and pitfalls of using tissue concentrations in clinical studies in IBD.

## 2. The Methods of Sampling for Obtaining Tissue Concentrations

There are various methods of measuring tissue concentrations in IBD such as whole tissue sampling, matrix-assisted laser desorption/ionization–mass spectrometric imaging, microdialysis, and fluorescent labeling of drugs. A summary of the advantages and disadvantages of each of the different sampling methods for obtaining tissue concentrations in IBD can be found in Table 1. 

### 2.1. Whole Tissue Sampling

Whole tissue sampling is the most frequently used method for obtaining tissue to measure concentrations of drugs. The concentrations are usually determined after homogenization or lyzation of biopsies obtained during endoscopy. It must be noted that tissues are composed of different pharmacokinetic compartments (interstitial fluid, cells, and, within cells, the various subcellular organelles) in which the drug is not necessarily distributed homogenously. After tissue homogenization, the free drug concentrations cannot be assessed in these various subcellular compartments because those compartments have been demolished by homogenization or lyzation. This can be a problem for drugs where timing of sampling may influence the local distribution in subcellular compartments, as tissue drug equilibrium might not have been achieved in these compartments yet [2]. An example is tofacitinib, which was shown to reduce inflammation in the colon but not in the ileum. This suggests the need for site-specific tissue sampling to accurately assess local drug concentrations in target tissues [4].

Furthermore, biopsies usually contain only mucosa and lamina propria, and thus may not fully represent the colonic tissue. Local differences in drug distribution within colonic tissue biopsies may exist for drugs [5]. Examples of methods that can be used to measure drug concentrations are liquid chromatography–mass spectrometry and enzyme-linked immunosorbent assay [6]. Another issue is the lack of guidelines and validation studies for bio-analysis of drug concentrations in tissues, in contrast to serum or plasma [7].

### 2.2. Matrix-Assisted Laser Desorption/Ionization–Mass Spectrometric Imaging (MALDI-MSI)

MALDI-MSI is an emerging cutting-edge tool for determining the spatial localization of molecules within tissues. It can generate distribution profiles (ion intensity maps) of dozens of molecular compounds, including endogenous molecules that have different mass/charge ratios, enabling molecular spatial distribution of a drug within biopsy [8]. Various successful applications of MALDI-MSI in IBD have been described previously [9]. Examples include proteomic profile analysis of inflamed and non-inflamed colonic biopsies [10] and identification of different UC and CD protein signatures in peripheral blood mononuclear cels [11]. Furthermore, MALDI-MSI can be used to quantify drugs and their metabolites [5]. However, quantification of drug concentration also has its pitfalls such as ion-suppression effects, variation of signal, and interference from matrix-related ions [12].

### 2.3. Microdialysis

Tissue cells are surrounded by interstitial fluid, which is the main component of the extracellular fluid in the human body. The tissue concentration of drugs can be measured by using microdialysis, a technique that involves the insertion of a probe into the tissue of interest. This probe can be continuously flushed with a tissue-compatible perfusion fluid and has a semipermeable membrane that allows drug uptake by passive diffusion. However, the semipermeable membrane allows the passage of molecules with a low molecular weight, and, therefore, the concentrations of unbound small molecules can be determined [13]. For larger proteins (like biologicals) different membranes exists that might allow the passages of these molecules [14]. The advantage of this method is that it allows continuous sampling within a certain time period. The drawback of this method is the invasive nature of the technique, which makes it difficult to use for the general IBD patient. It is currently used for postoperative monitoring of gastrointestinal organ viability [15].

### 2.4. Fluorescent Labeling of Drugs

Fluorescent labeling of drugs (FLD) is a technique used to measure local drug concentrations by tagging drugs with fluorescent markers, allowing for visualization and quantification in biological systems. Fluorochromes are substances that emit light after being excited by a specific wavelength of light. Commonly used fluorescent dyes include fluorescein, eosin, and rhodamine. FLD can be used for in vivo macroscopic imaging during endoscopy [16], but can also be combined with microscopy to provide cellular imaging and to investigate drug–target interactions [17]. Hence, the advantage of this method is that it allows for the spatial distribution of labeled molecules to be observed during endoscopy, as well as their cellular distribution within the tissue. A major disadvantage of this method is the need for the additional administration of fluorescent dyes. Moreover, the fluorescence signal can be influenced by several factors, such as interactions with other drugs, background tissue fluorescence, and limited tissue penetration [18,19,20].

## 3. Factors Influencing Tissue Concentrations

### 3.1. Physical-Chemical Properties of the Drug

The physical-chemical properties of a drug such as molecular weight, lipophilicity, and extent of ionization, and cellular transport can influence its pharmacokinetical properties [21,22]. The rate of diffusion is inversely proportional to the molecular weight of a drug, where high-molecular-weight drugs have low permeability. Small-molecule drugs with a molecular weight < 200 g/mol can permeate through tight junctions between intestinal cells via paracellular passive diffusion. Both in ulcerative colitis (UC) and in Crohn’s disease (CD), destruction of tight junctions increases the permeability of the larger molecule [23]. The lipophilicity is also an important factor that influences solubility, permeability and metabolism [24]. It is usually expressed as Log P (partition coefficient), which is the ratio of distribution of a drug in a mixture of a lipophilic and hydrophilic solvent. For lipophilic drugs (Log P > 3), the dissolution and solubility in the gastrointestinal fluids are often the rate-limiting factor for drug absorption as only the dissolved (ionized) part of a drug can permeate through the cell membrane. Changes in gastrointestinal transit times, reduced gastrointestinal volumes and a reduced intestinal absorptive area might all affect the gastrointestinal bioavailability of a drug and can occur in the changed intestinal physiology caused by IBD [23]. The degree of ionization influences both the solubility and the permeability of drugs and, subsequently, the rate of drug absorption. The rate of ionization depends on the pKa (acid-dissociation constant) of an individual drug. Weak bases are protonated in lower pH levels (pH ≤ 4), and are therefore soluble in gastric juice (pH ~1–2). When the soluble drug arrives in the duodenum, the higher pH results in a supersaturated state of the drug. The unprotonated form of the drug diffuses through the cellular membrane. More protonated forms of the drug become unprotonated due to equilibrium as described by the Henderson–Hasselbalch equation [25]. In CD and UC, the pH of the stomach might be elevated, and, therefore, solubilization of weak bases may be decreased [23]. Weak acids are more soluble on higher pH values. This may hinder drug permeability because only the unionized fraction of the drug is able to pass the cell membrane [26]. Furthermore, the free drug concentration of acidic drugs is higher in cytosolic compartments, whereas for basic drugs, drug concentrations might be higher in lysosomal or mitochondrial. This so-called ion trapping also depends on the cell type since the subcellular compartments of different cell types have different volumes and pH values [27,28]. Therefore, drug-specific pharmacochemical properties have a major impact on tissue concentrations in biopsies.

### 3.2. Timing of Sampling

For clinical and research purposes, the ratio between serum and tissue concentrations of a drug (tissue penetration ratio) can be highly relevant. Some aspects should be taken into account, however. The concentration–time curve can differ between serum and tissue levels due to systemic hysteresis (i.e., when tissue concentrations rise, subsequently serum concentrations decrease). In the fields of microbiology, neurology, and pulmonology, tissue penetration has been extensively studied [2,7,12]. In principle, peak tissue concentrations are in general lower and occur later compared to peak serum concentrations. Furthermore, tissue trough concentrations tend to be higher compared to serum trough concentrations. The measured tissue-penetration ratio may appear low when tissue samples are taken shortly after drug administration, but may appear higher if there is a longer lag time between tissue sampling and drug administration (depending on T_0.5_) [7]. Tissue sampling should therefore occur when a steady state concentration of the drug is reached, in both tissue and serum. To determine the number of dosages needed to establish a steady state, repeated sampling of serum and tissue concentrations might be needed. In IBD, this is especially challenging because tissue samples can only be obtained by endoscopy, and therefore repeated invasive sampling is cumbersome. Serum steady-state concentrations for infliximab, adalimumab, ustekinumab, and vedolizumab are achieved after approximately 14, 20, 16, and 6 weeks, respectively. Various factors may contribute to anti-monoclonal antibody availability. Factors include anti-drug antibodies, albumin levels and concomitant use of other medications [29]. The so-called ‘antigen sink’ theory might also contribute to monoclonal antibody clearance. Patients with severe inflammation might have higher TNF-alpha burden and thus tend to have a higher fraction of monoclonal antibody clearance. Therefore, these patients are prone to suboptimal response [30,31]. For azathioprine, methotrexate, and tofacitinib, time to steady state has been found of 4–6 weeks [32,33], 6–8 weeks [34] and 24–48 h [35]. The time until steady-state concentrations have been reached can be used to determine the timing of sampling.

### 3.3. IBD-Related Intestinal Inflammation May Affect Tissue Drug Concentrations

In IBD, intestinal inflammation may affect oral drug absorption through changes in transit time, local pH, drug permeability, metabolism, the microbiome [36], and the contents of the intestinal lumen [23]. In ulcerative colitis, the total gastrointestinal transit time is increased, especially in patients with severe disease [37,38]. The pH in the stomach is slightly increased in UC patients [39], whereas conflicting results have been published regarding colonic pH values in UC [23]. These differences in pH levels might affect absorption of oral small molecules. Moreover, phosphatidylcholine was strongly decreased in the colonic mucus of UC patients [40]. This decrease in phosphatidylcholine can increase intestinal permeability, which leads to increased absorption of certain drugs though the compromised barrier [41,42,43,44]. Furthermore, the surface hydrophobicity is affected by decreased levels of phosphatidylcholine, which can affect the absorption of either lipophilic or hydrophilic drugs [43,45]. The expression of cationic drug transporters, novel organic cation transporters (OCTN)1 and OCTN2, has been decreased in UC patients [46]. Drugs such as gabapentin, metformin, and verapamil are substrates for OCTN1 transporters which may affect the bioavailability of those drugs [47]. There is also decreased expression of P-glycoprotein, BRCP, and MPR2 transports [48]. Also, there is an increased expression of CYP3A4 and CYP2E1, but a decreased expression of CYP2C9 and UDP-glucuronic acid transferase [49,50]. These transporters affect bioavailability in a wide range of drugs such as digoxin, cyclosporin, and HIV protease inhibitors [51,52]. It is not clear to what extent UC affects transporter expression and drug bioavailability [51]. Small intestinal bacterial overgrowth (SIBO) was more prevalent in UC and CD patients [53,54]. SIBO may lead to unpredictable drug bioavailability because excess bacteria may directly metabolize drugs or interfere with drug transport [55]. In CD, gastric emptying is prolonged in the fed state [56], and the small intestinal transit time is also increased [57]. Orocaecal transit times were also found to be increased in CD patients [57]. Prolonged transit times may increase the absorption of drugs by allowing more time for dissolution and absorption in the small intestines [58]. However, the transit times in CD seem dependent on disease activity, and history of strictures and penetrating disease [59,60].

The gastric acid secretion is decreased, and the fecal osmolarity is increased [61,62]. Also, CYP3A4, CYP2D9, CYP1A1, CYP2B6, and UDP-glucuronic acid transferase were increased [49,50]. All these factors might affect tissue concentrations and their relationship with serum concentrations in IBD patients.

### 3.4. TNF-Alpha Inhibitors

Yoshihara et al. [63] reported a positive correlation between serum and tissue concentrations of anti-TNF, especially in uninflamed tissue. Furthermore, anti-TNF tissue concentrations correlated with the degree of endoscopic inflammation. The ratio of anti-TNF-to-TNF in tissue was highest in uninflamed areas and lowest in severely inflamed areas. Furthermore, patients with the highest active mucosal disease had a significantly higher serum-to-tissue ratio compared to patients in remission. These findings suggest that anti-TNF tissue concentrations may be used as a useful biomarker for remission in CD. This concurs with similar results published by Yarur et al. [64] and Choi et al. [65] who investigated tissue anti-TNF in IBD patients treated with IFX or ADA. Atreya et al. [66] used fluorescein isothiocynate-labeled ADA during endoscopy and then used confocal laser endomicroscopy to identify tumor necrosis factor-alpha-expressing cells.

### 3.5. Ustekinumab

Proietti et al. [67] conducted a prospective study investigating the correlation between ustekinumab (UST) concentrations in serum and tissue with clinical outcomes in CD patients. Follow-up was performed at week 16, during which UST serum and tissue concentrations were measured. They found that UST tissue concentrations correlated with UST serum concentrations (r^2^ = 0.35, *p* < 0.0001). Serum IL-23 concentrations negatively correlated with serum UST concentrations (r^2^ = 0.07, *p* = 0.04), but tissue IL-23 concentrations did not correlate with tissue UST concentrations (r^2^ = 0.01, *p* = 0.48). Additionally, tissue UST concentrations did not correlate with the severity of mucosal inflammation (r^2^ = 0.004, *p* = 0.47), although the tissue IL-23-to-UST ratio did positively correlate with mucosal inflammation (r^2^ = 0.048, *p* = 0.01). Furthermore, serum UST concentrations correlated with biochemical response (*p* = 0.01), while tissue UST concentrations did not correlate with clinical (*p* = 0.76), biochemical (*p* = 0.22), endoscopic (*p* = 0.31), or histological response (*p* = 0.75). Based on this study, the routine use of UST tissue concentrations appears to have no value in predicting response in CD patients.

### 3.6. Vedolizumab

Van den Berghe et al. [68] collected serum and tissue samples from 40 UC patients (20 endoscopic responders) after 14 weeks of VDZ treatment. There was a positive correlation between VDZ serum and tissue concentrations (ρ = 0.84, *p* < 0.0001), regardless of the macroscopic inflammation status. VDZ tissue concentrations were significantly lower in non-responders compared to responders (0.07 vs. 0.11 µg/mg, *p* = 0.04). In patients with adequate VDZ serum concentrations (>14.6 mg/L), the tissue VDZ concentrations were not significantly different between responders and non-responders (0.15 vs. 0.13 µg/mg; *p* = 0.92). Pauwels et al. [69] conducted a prospective study involving 37 IBD patients with active disease initiating vedolizumab treatment (VDZ). A positive correlation was found between tissue and serum VDZ concentrations at week 16 of treatment (r^2^ = 0.83; *p* < 0.0001). VDZ tissue concentrations inversely correlated with mucosal inflammation. The median VDZ concentrations in patients with no, mild, moderate, and severe endoscopic inflammation were 13.10 µg/mL, 10.30 µg/mL, 7.37 µg/mL, and 6.65 µg/mL, respectively, showing an inverse correlation between tissue VDZ concentration and endoscopic score (*p* = 0.06). Serum VDZ concentrations did not correlate with the severity of endoscopic inflammation (*p* = 0.32). VDZ tissue concentrations were associated with biochemical (*p* = 0.002) and endoscopic (*p* = 0.04) outcomes. Serum VDZ concentrations were associated with biochemical outcomes (*p* = 0.03), but not with endoscopic outcomes (*p* = 0.21). The findings of this study suggest that additional measurement of tissue VDZ concentrations may aid in therapeutic drug monitoring in VDZ-treated patients.

The pharmacokinetic and dynamic features of VDZ were studied by Ungar et al. [70] They performed a prospective study with 106 IBD patients who were treated with VDZ. In their study, clinical remission was achieved by 48% of patients at week 14 of treatment. No significant clinical outcomes were associated with medium serum VDZ, except for CRP-level. Anti-vedolizumab antibodies (AVA) did not correlate with clinical outcomes. Furthermore, they performed flow-cytometry analysis of peripheral blood memory T-cells which showed almost complete occupancy of α4β7 integrin (target of VDZ therapy), regardless of response status or serum level. Gabriëls et al. [71] used fluorescence molecular imaging (FMI) to visualize both macroscopic and microscopic distribution of intravenously administrated, fluorescently labeled VDZ (vedo-800 CW) in 43 IBD patients. In the dose-finding phase of the study, patients received an intravenous dose of 4.5 mg, 15 mg vedo800CW, or no tracer prior to endoscopy. In the target-saturation phase, patients received 15 mg vedo-800CW preceded by an unlabeled (sub)therapeutic dose of VDZ. In the dose-finding phase, FMI quantification showed a dose-dependent increase in vedo-800CW fluorescence intensity in inflamed tissue. They found that 15 mg of vedo-800CW had the most optimal differentiation between non-inflamed and actively inflamed tissue. Furthermore, in the target-saturation phase, the fluorescence was the highest in the subtherapeutic dose group (75 mg, 102 au {86–164}]) and was the lowest in the group of patients who received theirs after >14 weeks of therapy followed by 15 mg vedo-800CW (59 au {50–91}). This suggests that vedo-800CW binding was blocked by unlabeled VDZ, or can be interpreted as that a single therapeutic dose can already saturate the target tissue. They also performed ex vivo fluorescence microscopy in order to assess the distribution of vedo-800CW. Vedo-800CW had deep penetration in affected tissue samples and there was a heterogenous distribution. Furthermore, they reported binding between vedo-800CW and plasma cells, and intracellular presence of vedo-800CW in both eosinophils and macrophages.

### 3.7. Tofacitinib

Verstockt et al. [72] reported tissue concentrations in 30 UC patients treated with tofacitinib (TFC). They performed endoscopic assessment at baseline and 8–16 weeks after TFC initiation. There was a significant correlation between TFC tissue and serum concentrations (r = 0.92, *p* < 0.001), although tissue concentrations were significantly higher than serum concentrations (520.19 ng/g vs. 17.35 ng/mL, *p* < 0.001). Furthermore, TFC tissue concentrations were associated with endoscopic improvement at week 16 (*p* = 0.04).

### 3.8. Methotrexate

Van de Meeberg et al. [73] investigated methotrexate (MTX) concentrations in red blood cells (RBCs), peripheral blood mononuclear cells (PBMCs) and intestinal mucosa in CD patients. They reported a marked accumulation of MTX-PG_1–6_ in intestinal mucosal biopsies with interpatient variability, and found no correlation between treatment duration and concentration of MTX-PGs. In tissue biopsies, MTX-PG_1_ was the predominant species, whereas long chain MTX-PG_4–6_ (glutamates with the highest retention) were also prevalent. There were no significant differences in MTX-PGs in inflamed biopsies compared to non-inflamed biopsies. After discontinuation of therapy in three patients, the MTX-PG_1_ concentration dropped significantly in all biopsies, while MTX-PG_4–6_ was largely retained. In contrast to PMBCs, where all MTX-PG concentrations rapidly declined. This suggests that the MTX-PG concentration in PMBCs reflects MTX metabolite concentrations in the serum, while active metabolites may still be measurable in the intestinal mucosa after discontinuation of therapy.

## 4. Outlook and Future Perspectives

Based on the current evidence from available studies (see Table 2), tissue-concentration measurements have no established role in clinical practice. However, they may provide valuable insights into the pharmacology of drugs in IBD and could play a more prominent role in future clinical trials. Despite this potential, it is unlikely that tissue concentrations will be routinely used in clinical care due to challenges related to validation. This view is primarily supported by microbiological studies, which indicate that correlating tissue concentrations with blood sample levels is unwarranted and could potentially harm patient care [2].

## 5. Conclusions

Investigating drug tissue concentrations can enhance our understanding of IBD drug pharmacology and the impact of local drug concentrations on inflammation. Continued research and methodological advancements will be essential to reveal the full potential of tissue-concentration measurements in understanding drug pharmacology. To date, the routine use of tissue-concentration measurements in IBD is not yet established.

## Figures and Tables

**Table 1:** The advantages and disadvantages of using tissue concentrations in clinical IBD studies. Matrix-Assisted Laser Desorption/Ionization–Mass Spectrometric Imaging (MALDI-MSI).

Advantage	Pitfalls
General ▪ Enhance understanding between tissue concentrations and local inflammation mediators. ▪ Possibility to enhance therapeutic drug monitoring by correlating serum and tissue drug concentrations.	General ▪ Tissue concentrations may be affected by altered intestinal tissue structure and permeability due to increased IBD-related inflammation. ▪ IBD disturbs intestinal physiology, which affects local drug concentrations ▪ Physical-chemical property changes may affect tissue concentrations of the drug. ▪ Timing of sampling of tissue concentrations ▪ No standardized guidelines or validation studies for bioanalysis of drugs in tissues.
Whole tissue sampling ▪ Correlation between mucosal inflammation and tissue drug concentration.	Whole tissue sampling ▪ No assessment of free drug concentrations in subcellular compartments due to tissue homogenization or lyzation.
Microdialysis ▪ Continuous sampling within a certain time period. ▪ Drug concentrations can be measured in specific intestinal locations.	Microdialysis ▪ Invasive measurement technique. ▪ Analysis of only unbounded molecules; semipermeable membrane only allows passage of small molecules.
MALDI-MSI ▪ Spatial distribution of molecules in biopsy material, which allows spatial analysis of drug concentrations. ▪ Quantification of drug concentrations.	MALDI-MSI ▪ Quantification of drug concentrations needs validation study.
Fluorescent-labeled drugs ▪ Allows for spatial distribution of labeled molecules during endoscopy and tissue. ▪ Investigation of drug–target interactions in specific immune cell subsets.	Fluorescent-labeled drugs ▪ Needs specific intravenous administration of fluorescently labeled drug. ▪ Intrinsic fluorescence of local tissue. ▪ Toxicity of fluorophores.

**Table 2:** Overview of studies that used tissue concentrations of IBD drugs. IFX: infliximab, ADA: adalimumab CD: Crohn’s disease, UC: ulcerative colitis. UST: Ustekinumab, VDZ: vedolizumab, MTX: methotrexate, TFC: tofacitinib, TNF: tumor necrosis factor. Supporting evidence was defined as whether main results support the use of tissue concentrations in IBD.

Author	Drug	IBD	Main Result	Supporting Evidence
Yoshihara [63]	IFX + ADA	CD	▪ Positive correlation between serum and tissue concentrations of anti-TNF, especially in uninflamed tissue. ▪ Anti-TNF tissue concentrations correlated with endoscopic inflammation. ▪ Anti-TNF-to-TNF ratio in tissue was highest in uninflamed areas and lowest in severely inflamed areas. ▪ Highest active mucosal disease had a significant higher serum-to-tissue ratio compared to patients in remission	Yes
Yarur [64]	IFX + ADA	CD + UC	▪ Correlation anti-TNF serum and tissue concentrations, especially in uninflamed tissue, but not in inflamed tissue. ▪ Anti-TNF in tissue correlated with endoscopic inflammation, except for severe inflammation. ▪ Anti-TNF-to-TNF ratio in tissue was highest in uninflamed areas and lowest in severely inflamed areas.	Yes
Choi [65]	Infliximab	UC	▪ Positive correlation between serum and tissue concentrations of anti-TNF, only in uninflamed tissue. ▪ Neither plasma nor tissue TNF-α concentrations were associated with histologic disease activity.	No
Atreya [66]	IFX + ADA	CD	▪ High numbers of mTNF+ cells showed significantly higher short-term response rates at week 12 with anti-TNF therapy as compared to patients with low amounts of mTNF+ cells.	Yes
Proietti [67]	Ustekinumab	CD	▪ UST tissue concentrations correlated with UST serum concentrations. ▪ Serum IL-23 concentrations negatively correlated with serum UST concentrations, but tissue IL-23 concentrations did not correlate with tissue UST concentrations. ▪ UST concentrations did not correlate with the severity of mucosal inflammation. ▪ Tissue IL-23-to-UST ratio did positively correlate with mucosal inflammation. ▪ Serum UST concentrations correlated with biochemical response. ▪ Tissue UST concentrations did not correlate with clinical, biochemical, endoscopic, or histological response.	No
Van den Berghe [68]	Vedolizumab	UC	▪ Positive correlation between VDZ serum and tissue concentrations, regardless of the macroscopic inflammation status. ▪ VDZ tissue concentrations were significantly lower in non-responders compared to responders. ▪ In patients with adequate VDZ serum concentrations (> 14.6 mg/L), the tissue VDZ concentrations were not significantly different between responders and non-responders.	No
Pauwels [69]	Vedolizumab	CD + UC	▪ A positive correlation was found between tissue and serum VDZ concentrations at week 16. ▪ VDZ tissue concentrations inversely correlated with mucosal inflammation. ▪ Inverse correlation between tissue VDZ concentration and endoscopic score. ▪ Serum VDZ concentrations did not correlate with the severity of endoscopic inflammation. ▪ VDZ tissue concentrations were associated with biochemical and endoscopic outcomes. ▪ Serum VDZ concentrations were associated with biochemical outcomes, but not with endoscopic outcomes.	Yes
Ungar [70]	Vedolizumab	CD + UC	▪ No significant clinical outcomes were associated with medium serum VDZ, except for CRP-level. ▪ Anti-vedolizumab antibodies did not correlate with clinical outcomes. ▪ Flow-cytometry analysis of peripheral blood memory T-cells showed almost complete occupancy of α4β7 integrin (target of VDZ therapy), regardless of response status or serum level.	No
Gabriëls [71]	Vedolizumab	CD + UC	▪ The most optimal differentiation between non-inflamed and actively inflamed tissue occurred with 15 mg of vedo-800CW. ▪ Fluorescence was the highest in the subtherapeutic dose group (75 mg, 102 au {86–164}) and was the lowest in the group of patients who received theirs after >14 weeks of therapy followed by 15 mg vedo-800CW (59 au {50–91}). ▪ Vedo-800CW had deep penetration in affected tissue samples and there was a heterogenous distribution. ▪ Binding between vedo-800CW and plasma cells, and intracellular presence of vedo-800CW in both eosinophils and macrophages.	No
Verstockt [72]	Tofacitinib	CD + UC	▪ Significant correlation between TFC tissue and serum concentrations, although tissue concentrations were significantly higher than serum concentrations. ▪ TFC tissue concentrations were associated with endoscopic improvement at week 16.	Yes
Van de Meeberg [73]	Methotrexate	CD	▪ Marked accumulation of MTX-PG1-6 in intestinal mucosal biopsies with interpatient variability; no correlation was found between treatment duration and concentration of MTX-PGs. ▪ In tissue biopsies, MTX-PG1 was the predominant species, whereas long chain MTX-PG4-6 (glutamates with the highest retention) were also prevalent. ▪ No significant differences in MTX-PGs in inflamed biopsies compared to non-inflamed biopsies. ▪ After discontinuation of therapy in three patients, the MTX-PG1 concentration dropped significantly in all biopsies, while MTX-PG4-6 was largely retained.	No

## Data Availability

Not applicable.
